# The Tortuous Road to Ending Preventable Maternal Deaths in Nigeria

**DOI:** 10.5334/aogh.5197

**Published:** 2026-04-16

**Authors:** Gabriel Dogbanya

**Affiliations:** 1 Doctoral student, Maternal and Child Health, Family Science Department, University of Maryland, College Park, USA

**Keywords:** Maternal Death Nigeria

## Abstract

Nigeria continues to bear a disproportionate share of the global burden of maternal mortality despite decades of national and international commitments to its reduction. This viewpoint examines the tortuous and unstable trajectory of efforts to end preventable maternal mortality in Nigeria, noting a modest decline from extremely high levels exceeding 1000 maternal deaths per 100,000 live births. A shift that signals incremental progress, however, remains fragile, nonlinear, and vulnerable to reversal due to recurrent programmatic setbacks and weak health system resilience. The article situates Nigeria’s experience within the global ending preventable maternal mortality framework, analyzing the continued relevance of its five strategic objectives in the Nigerian context. It reviewed key national initiatives from the Midwives Service Scheme to the widely acclaimed “Abiye” program in Ondo State—highlighting how political transitions, funding instability, and weak institutionalization have undermined continuity and scale-up. It argues that Nigeria’s fluctuating maternal mortality trends are closely linked to inconsistent coverage, quality, and sustainability due to discontinuity driven by political turnover. It calls for the elimination of all barriers to care, functional emergency obstetric care services, governance, and accountability through the Maternal and Perinatal Death Surveillance and Response program.

## Introduction

Nigeria has always been part of the global effort to address the high maternal mortality ratio (MMR) in developing countries, from the launch of the Safe Motherhood Initiative in 1987 through to the current Sustainable Development Goals (SDGs) agenda, yet the lives of many women, often in their prime, are prematurely lost through avoidable circumstances during pregnancy and childbirth [1, 2]. Ending preventable maternal mortality (EPMM) remains one of the most persistent public health challenges in Nigeria. Over the past two decades, the country’s MMR has followed a tortuous and uneven trajectory, with estimates exceeding 1000 maternal deaths per 100,000 live births—levels categorized as extremely high and emblematic of deep structural vulnerabilities within the maternal health ecosystem and reflects the complex interplay of health system weaknesses, socioeconomic inequities, policy discontinuities, and contextual shocks [3, 4]. Currently, Nigeria still leads the field with the highest number of maternal deaths with 993 per 100,000 live births (UI 718–1540), in 2023, accounting for more than a quarter (28.7%) of all estimated global maternal deaths and approximately 75,000 deaths [3]. This reflects a modest but notable shift from previously reported extremely high maternal mortality levels [5–7]. While this reduction signals progress, it is neither linear nor secure and instead reflects incremental gains achieved amid recurrent setbacks, uneven subnational performance, and fragile health system resilience.

Globally, efforts to reduce maternal mortality continue, with the MMR declining by 40.0% between 2000 and 2023 from 328 (UI 308–352) to 197 (UI 174–234) per 100,000 live births [3]. The benchmark remains SDG 3, particularly target 3.1, which aims to reduce the global MMR to fewer than 70 per 100,000 live births by 2030 [8]. However, the wide gap between this target and Nigeria’s current reality highlights deep inequities in healthcare delivery and underscores the urgent need for comprehensive, context-specific maternal health interventions [9].

Nigeria’s fluctuating maternal mortality trends are closely associated with inconsistent coverage, quality, continuity, and scalability of key maternal health interventions, including established programs and partnerships [1, 10–13]. Frequent political turnover and weak institutionalization contribute to program interruptions, producing the “tortuous” trajectory observed in maternal mortality trends [1, 14]. Solanke and colleagues, in their review of maternal and child health outcomes in Nigeria from 1978 to 2023, identified institutional quality as a major determinant of outcomes [15], while Kuti et al. observed that many programs were not sustained and often ended with the tenure of the government that initiated them [1]. These systemic challenges result in inadequate financing for health and healthcare and the suboptimal use of available resources to procure and deliver services [16]. Consequently, program disruptions occur, leading to discontinuities in coverage and quality and slowing progress toward reducing maternal mortality [1]. These gaps are further compounded by delayed care-seeking, persistent urban–rural inequities, workforce shortages, and sociocultural barriers [17–19]. Although periodic policy reforms and donor-supported initiatives have generated short-term improvements, their long-term impact has often been constrained by weak implementation fidelity, limited institutionalization, and insufficient integration into broader health system strengthening efforts [1].

As the global development agenda transitioned from the Millennium Development Goals to the SDGs, the target of reducing maternal mortality to fewer than 70 per 100,000 live births by 2030 emerged from the 2014 World Health Organization (WHO) consensus statement on targets and strategies for EPMM [20, 21]. The five strategic objectives articulated under the EPMM framework remain highly relevant within the Nigerian context today.

- Address inequities in access to and quality of sexual, reproductive, maternal, and newborn health care: Inequitable access to maternal health services remains a persistent challenge, with Nigeria ranking among the poorest globally in both access to and quality of healthcare services [22, 23]. Socioeconomic disadvantage, ethnic marginalization, and geographic barriers disproportionately limit service utilization among underserved populations. Addressing these inequities is essential to reducing the elevated maternal morbidity and mortality risks borne by these groups.
- Ensure universal health coverage for comprehensive sexual, reproductive, maternal, and newborn health care: In Nigeria, household out-of-pocket expenditure remains the predominant mode of healthcare financing, in stark contrast to financing arrangements in more developed settings [24]. Evidence indicates that the National Health Insurance Scheme (NHIS) improves access to and utilization of maternal and child health services and is associated with reductions in maternal mortality [25]. Expanding the NHIS toward effective universal health coverage is, therefore, critical for improving maternal and newborn health outcomes.
- Address all causes of maternal mortality, reproductive and maternal morbidities, and disabilities: Although hypertensive disorders of pregnancy, obstetric hemorrhage, and sepsis remain the leading causes of maternal mortality [1, 2, 4, 26], ending preventable maternal deaths requires a comprehensive approach that addresses the full spectrum of direct and indirect causes. This demands a sustained high index of clinical suspicion and timely management across the continuum of maternal care.
- Strengthen health systems to respond to the needs and priorities of women and girls: Achieving universal health coverage and sustaining essential health services depend on the presence of a functional and resilient health system [22]. Comprehensive implementation of all major health-system building blocks is therefore required, as demonstrated by the success of the “Abiye” program in Ondo State [27].
- Ensure accountability to improve quality of care and equity: Accountability is essential if Nigeria’s strained health system is to achieve meaningful and sustained improvements. As experts have noted, the central challenge is not solely the availability of skilled clinicians, advanced therapies, or new technologies—important as these are—but the presence of effective governance [28, 29]. The Maternal and Perinatal Death Surveillance and Response (MPDSR) program was designed to promote accountability at both institutional and community levels. However, implementation remains limited nationwide [30, 31].

Over the years, Nigeria has implemented several targeted interventions aimed at reducing maternal deaths, with varying degrees of success [1]. In 2007, the Integrated Maternal, Newborn and Child Health (IMNCH) strategy was introduced and aimed to promote life-saving interventions across the pregnancy and childbirth period [4]. Another major national initiative was the Midwives Service Scheme (MSS), launched in 2009 and run through 2015, by the Federal Government in collaboration with state and local governments [1, 32]. The scheme deployed midwives to underserved rural communities in an audacious effort to address maternal mortality nationwide. However, like many interventions in Nigeria, the scheme’s impact was constrained by persistent funding challenges, patchy and poor implementation, limited coverage of only 10% of local government areas in Nigeria, and its discontinuation following changes in political leadership [1, 4]. The Basic Healthcare Provision Fund (BHCPF), created under the National Health Act (2014), funds a basic minimum package of health services with primary health care strengthening and services relevant to maternal (and child) health [33]. It is a program deployed by the federal government of Nigeria to bypass some of the pitfalls of existing health financing arrangements [34]. However, a review across six states in Northern Nigeria: Bauchi, Borno, Kaduna, Kano, Sokoto, and Yobe found suboptimal implementation of the BHCPF in at least one thematic area across all states [33]. At the subnational level, the “Abiye” Safe Motherhood Initiative, piloted in Ondo State, stands out as a transparent, well-documented, and highly regarded model for reducing maternal mortality in rural and hard-to-reach populations [1, 27, 35]. The program tracked every pregnant woman from conception to delivery, removed financial barriers completely, and improved the supply and demand side of services. Evaluations reported substantial reductions in maternal and perinatal deaths, and the initiative received commendation from the World Bank and the Bill and Melinda Gates Foundation as a replicable model for other low-resource settings [36–38]. The Abiye program significantly improved maternal health outcomes, reducing the maternal mortality ratio by 84.9% from 745 per 100,000 live births in 2009 to 112 per 100,000 live births in 2016 [39], while substantially increasing antenatal care utilization and facility-based deliveries, which increased by 29 percentage points [36]. Sadly, the program was not sustained with a change in government [1]. Similarly, other state governments, including Lagos, Kaduna, Katsina, Kano, and others, introduced maternal health interventions such as the establishment and expansion of maternal and child health centers [40–44]. In Jigawa State, the partnership with Médecins Sans Frontières (MSF) in Jahun continues to yield measurable reductions in maternal mortality, highlighting the role of partnership in this quest [45]. Nevertheless, while these initiatives demonstrate what is achievable, isolated successes, particularly those limited to a single region, state, district, or facilities, remain insufficient to fundamentally alter the national maternal mortality landscape without national institutionalization.

Preventing maternal mortality in Nigeria requires a strategic recalibration that goes beyond episodic gains toward stabilizing progress through consolidation, equity, and scalability across regions. While several initiatives aimed at reducing maternal mortality have been proposed [2, 40, 46], the following recommendations are apt based on identified gaps highlighted by recent reports. First, policy inconsistency must be addressed. Program disruptions resulting from political transitions undermine sustained progress in reducing maternal mortality. Ending preventable maternal deaths should be pursued as a long-term, implementation-focused goal insulated from political turnover. Proven interventions should be revived, strengthened, and scaled, supported by legislation that guarantees continuity regardless of the political climate. Second, Nigeria must prioritize the removal of barriers that limit the utilization of hospitals and formal health facilities. These include financial constraints, poor transportation systems, and persistent cultural and religious misconceptions surrounding facility-based delivery [47]. Evidence shows that, despite the availability of effective interventions, health-service utilization remains suboptimal in some settings [4, 48]. Addressing this paradox requires coordinated action among researchers, policymakers, and other stakeholders, with meaningful engagement of community partners to identify and dismantle context-specific deterrents to care-seeking. Even a strengthened health system and resilient intrapartum care package yield limited benefits when facility utilization is low [46]. Empirical evidence highlights the consequences of delayed care-seeking. Anumodu et al. reported that among women who died from hypertensive disorders of pregnancy, 93% presented late in critical condition, 42.5% had no antenatal care, and residence more than 5 km from a hospital increased mortality risk. Similarly, among women with postpartum hemorrhage, 50% presented late after referral, and 83% of those with severe outcomes arrived critically ill and in shock [4]. Third, Nigeria must eliminate delays in accessing care by ensuring that all hospitals and delivery facilities maintain dedicated emergency obstetric care (EmOC) units with 24-hour standby teams, supported by standard operating guidelines, laboratory services, and efficient referral systems [49]. A recent systematic review of literature revealed widespread inadequacies in EmOC service availability across Nigerian public healthcare facilities [50]. Fourth, bureaucratic bottlenecks that often lead to system inefficiency by hindering communication and coordination among communities, local, state, and federal governments, as well as international partners, must be dismantled [51]. The MPDSR program should be fully empowered to generate reliable, actionable data.

In conclusion, Nigeria’s maternal mortality trajectory reflects modest gains constrained by systemic instability. While effective interventions exist, weak institutionalization, funding volatility, and political discontinuity have limited their impact. Achieving irreversible reductions now requires shifting from short-term gains to resilient, system-wide solutions that ensure equitable, continuous, high-quality maternal care—affirming the Abiye program’s principle that “pregnancy should never again be a death sentence [52].”
